# Upstream – News in Genomics

**DOI:** 10.1002/cfg.172

**Published:** 2002-06

## Abstract

In recent months a bumper crop of genomes has been completed, including the fission yeast (*Schizosaccharomyces pombe*) and rice (*Oryza sativa*). Two large-scale studies of *Saccharomyces cerevisiae* protein complexes provided a picture of the eukaryotic proteome as a network of complexes. Amongst the other stories of interest was a demonstration that proteomic analysis of blood samples can be used to detect ovarian cancer, perhaps even as early as stage I.


**Comparative and Functional Genomics**
*Comp Funct Genom* 2002; **3**: 221–225.
Published online 2 May 2002 in Wiley InterScience (www.interscience.wiley.com). **DOI:** 10.1002/cfg.172

**Feature**

### Upstream – news in genomics

### Abstract

**In recent months a bumper crop of genomes has been completed, including the fission yeast (*Schizosaccharomyces pombe*) and rice (*Oryza sativa*). Two large-scale studies of *Saccharomyces cerevisiae* protein complexes provided a picture of the eukaryotic proteome as a network of complexes. Amongst the other stories of interest was a demonstration that proteomic analysis of blood samples can be used to detect ovarian cancer, perhaps even as early as stage I. Copyright © 2002 John Wiley & Sons, Ltd.**

### Genome sequencing

At the end of December, a Chinese consortium surprised the international rice community by reporting a **draft assembly of a rice (*Oryza sativa*) genome** in the *Chinese Science Bulletin* (Yu *et al*., 2001). This team selected the subspecies *indica*, rather than *japonica* (the subspecies chosen by International Rice Genome Project consortium, Monsanto and Syngenta). This subspecies is dominantly planted in Asia and other parts of the world and is a parent of the super-hybrid strain that is being used in efforts to solve the food supply problem in China. Tests against finished *indica* and *japonica* BAC sequences, a *japonica* physical map and *japonica* ESTs, cDNAs and unigene clusters all indicated 95%, or better, coverage of the genome. All draft sequences above 1–2 kb are available from GenBank and at **http://www.genomics.org.cn**. The draft was announced at Plant, Animal and Microbe Genomes X in January (see Wixon and Dicks, 2002 for a detailed report), at which time the manuscript was under assessment for publication in *Science*. Rumours that Syngenta planned to publish their ***japonica* genome** in the same issue of *Science*, without public release of the data, sparked a row (Butler, 2002). The two papers were finally published in early April (Yu *et al*., 2002; Goff *et al*., 2002). Syngenta have 42 000 sequence contigs ranging from 5 kb to ~150 Kb, which are estimated to cover more than 99% of the rice genome and greater than 99% of rice genes, the majority of them are mapped. The Syngenta data has not been deposited in the public databases, but is available to academics from their website (**http://portal.tmri.org/rice/**) for BLAST searching, or downloading at a rate of 100 kb per week. The whole genome can be obtained on a CD ROM, after signing a Public Access Agreement. There is also an agreement to provide access for researchers at commercial organisations. A striking difference between the two papers is that Syngenta predict 33 000 to 50 000 genes, whilst the Chinese group predict 53 000 to 65 000 genes. Possible reasons for this are the higher stringency used by Syngenta (e.g:, they discounted any hypothetical genes that were less than 300 base pairs in size), and the greater time available to them for manual annotation.

In the January 22$^{nd}$ issue of *PNAS*, a Japanese team published the **genome sequence of *Clostridium perfringens* strain 13** (Shimizu *et al*., 2002) and Fitz-Gibbon *et al*. (2002) reported on their **genome sequence for *Pyrobaculum aerophilum***. *Clostridium perfringens* is a Gram-positive anaerobic spore-forming bacterium. It causes life-threatening gas gangrene and mild enterotoxaemia in humans, although it is present in the normal intestinal flora of humans and animals. Its low G+C content (28.6%), ~3 Mb genome includes 2660 protein coding regions and 10 rRNA genes. Twenty new putative virulence factor genes, and five hyaluronidase genes which will contribute to virulence, were identified. Four members of the two-component VirR/VirS regulon, that coordinately regulates the pathogenicity of *C. perfringens*, were found. *C. perfringens* lacks tricarboxylic acid cycle and respiratory chain enzymes, and obtains various essential materials from the host by producing a selection of degradative enzymes and toxins, resulting in massive destruction of the host tissues.

*Pyrobaculum aerophilum* is a facultatively aerobic, nitrate-reducing, hyperthermophilic crenarchaeon. The team's analysis of its 2.2 Mb genome confirmed that it lacks 5′ untranslated regions in its

Copyright © 2002 John Wiley & Sons, Ltd.

mRNAs, which would imply that it does not use a ribosome-binding site (Shine-Dalgarno)-based mechanism for initiation of translation at the 5′ end of transcripts. Looking at mononucleotide repeat-tracts, they saw that runs of Gs (or Cs) are highly unstable, which is to be expected in this mismatch repair deficient organism. This supports the suggested 'mutator' phenotype implied by an independent study on mutation rates. They also found some possible explanations for *P. aerophilum's* surprising intolerance to sulphur.

The **genome sequence of *Ralstonia solanacearum* (strain GMI1000)**, a soil-borne plant pathogen with a global distribution and wide host range, was published in *Nature* on January 31st (Salanoubat *et al.* 2002). The genome is organized into two replicons: a 3.7 Mb chromosome and a 2.1 Mb megaplasmid; there is evidence for the acquisition of genes through horizontal gene transfer on both. Many genes with a potential role in pathogenicity were found, including a large number of putative attachment factors. More than 40 candidate type III secreted effector proteins were identified. Comparing these with other genomes suggests that bacterial plant pathogens and animal pathogens harbour distinct arrays of specialized type III-dependent effectors.

On the 11th February an international consortium (composed of researchers from the Sanger Institute, TIGR, the US NMRC, and Stanford University) gave delegates at the 2nd ASM and TIGR Conference on Microbial Genomes a first look at the **genome of *Plasmodium falciparum***, the most important malaria parasite. The 30 Mb, AT-rich genome sequence is almost complete; they hope to publish the results this summer. Most of the data is already in the public databases and is also available from the consortium website (PlasmoDB 16). Both the Sanger Institute and TIGR have already started work on the genomes of other Plasmodium species, most of which could be finished by the end of this year.

On the 21st February the **complete genome sequence of the fission yeast, *Schizosaccharomyces pombe*** was published in *Nature* by an international consortium (Wood *et al.*, 2002). It has 4824 genes (the lowest gene count yet for a eukaryote), 43% of which have introns, and the upstream regions of genes are longer than in *S. cerevisiae* (see Wixon, 2002, for a detailed report). There are 50 genes with significant homology to human disease genes; half of these are cancer related. The team have identified highly conserved genes important for eukaryotic cell organization, which may have originated with the appearance of eukaryotic life. The sequence data is available for BLAST searching and downloading from: **http://www.sanger.ac.uk/Projects/S_pombe/**. Annotation and further information on all *S. pombe* genes can be obtained at: **http://www.genedb.org/genedb/pombe/index.jsp**.

On the 1st March the Sanger Institute announced that the 4.4 Mb **genome of *Mycobacterium bovis* strain AF2122/97 (spoligotype 9)** was finished. *M. bovis* is the major cause of tuberculosis in cattle and other animals, and can be transmitted to man. The chosen strain (taken from a cow with lesions in its lungs) was sequenced in collaboration with the Unit de Génétique Moléculaire Bactérienne, at Institut Pasteur and the UK Veterinary Laboratories Agency. The *M. bovis* genome is ~4.3 Mb, with a G+C content of 65.63%. The data is available to download or for BLAST searching (Sanger *M. bovis* page 18), although the team remind users that it is to be considered preliminary until the annotation is completed and the sequence is published.

Two papers published in March and April continued the debate on **Celera's whole genome shotgun (WGS)** approach. In the first paper, Waterston *et al.* (2002) assert that the way that Celera shredded the public HGP data to combine it with their WGS data to make their assembly (Venter *et al.*, 2001) practically guaranteed that the data would reassemble correctly, implicitly preserving much of the HGP assembly information. They provide simulations demonstrating this effect using HGP data from chromosome 22 (International Human Genome Sequencing Consortium, 2001) and conclude that the Celera assembly is not a true test of the WGS approach. In the second paper, Myers *et al.* (2002) refute these arguments, stating that due to the number of repeats across the genome, a simulation using chromosome 22 data is 100 times easier than the whole genome assembly that they undertook. They report that rerunning the chromosome 22 simulation, allowing 94% identity in overlaps, rather than 100%, results in a larger number of contigs than in the starting assembly, rather than an 'implicit reassembly'. This fracturing of chromosome 22 worsens when the simulation is run in the context of the whole genome data. They then make a comparison of the two genome assemblies, which indicates that there is a larger proportion of

Copyright © 2002 John Wiley & Sons, Ltd. 

non-repetitive sequence that is unique to the Celera assembly than is unique to the HGP assembly.

### Comparative genomics

In the February 5th Issue of *PNAS* a team from the Department of Microbiology and Molecular Genetics at Harvard Medical School published a **gene content comparison of several strains (including the 7th pandemic El Tor O1 strain N16961) of *Vibrio cholerae*** (Dziejman *et al.*, 2002) using an array including over 93% of predicted N16961 genes. They were surprised to see a high degree of conservation among the strains they tested, but they did identify genes unique to all pandemic strains, and genes specific to the 7th pandemic El Tor and related O139 serogroup strains. They suggest that these genes could be responsible for promoting the establishment of endemic disease in previously cholera-free locations.

### Functional genomics

A **genome-wide search for *Haemophilus influenzae* genes required for growth or survival** was published in the January 22nd issue of *PNAS* by Akerley *et al.* (2002). Using a high-density transposon mutagenesis strategy, 259 ORFs of unknown function were designated as putative essential genes. 12 of these proteins were shown to have homologues in *Mycobacterium tuberculosis* but none in *Saccharomyces cerevisiae*, making them potential targets for antimicrobial therapeutics. Three of these genes are essential for viability in other bacteria. Looking at the homologues for the putative essential genes in other microorganisms, they identified proteins involved in pathways that appear to be essential only in certain bacteria, however, they point out that the underlying experiments were done using different culture conditions and media.

In the February 5th issue of *PNAS* a group from the Cold Spring Harbor Laboratory (Paddison *et al.* 2002) demonstrated **silencing of gene expression by RNAi upon treatment of cultured murine cells with long dsRNAs (500 nt)**. RNAi has previously been shown to be a natural response in plants, *Caenorhabditis elegans*, *Drosophila*, and trypanosomes, however, in mammalian cells, only treatment with 22 nt RNAs (that mimic those produced in the first step of the silencing process) had been shown to elicit the response. They further showed that enforced expression of long, hairpin dsRNAs induced stable gene silencing. The ability to create such stable 'knock-down' cell lines will enable the examination of phenotypes that develop over long time periods, and will hopefully form the basis for using RNAi in phenotype-based, forward genetic selections.

### Proteomics

In the January 10th issue of *Nature*, a team from Cellzome, with collaborators at EMBL, reported on a large-scale study of **multiprotein complexes in *Saccharomyces cerevisiae*** using tandem-affinity purification (TAP) and mass spectrometry (Gavin *et al.*, 2002). They tagged 1739 genes (including 1143 with human orthologues) and purified 589 protein assemblies. Their analysis of the assemblies defined 232 distinct multiprotein complexes and allowed them to propose new cellular roles for 344 proteins, 231 of which had no previous functional annotation. Comparing yeast and human complexes showed that conservation across species extends from single proteins to their molecular environment. Their results provide an outline of the eukaryotic proteome as a network of protein complexes. They propose that this higher-order map contains fundamental biological information and offers the context for a more reasoned and informed approach to drug discovery.

In the same issue, a similar study by a group from MDS Proteomics, the Samuel Lunenfeld Research Institute, and the University of Toronto was published (Ho *et al.*, 2002). They also used a mass spectrometric approach, called high-throughput mass spectrometric protein complex identification (HMS–PCI), to identify **protein complexes in *Saccharomyces cerevisiae***. They used 10% of predicted yeast proteins as baits, and detected 3617 associated proteins, covering 25% of the yeast proteome. Amongst the many protein complexes identified were several new interactions within signalling pathways and in the DNA damage response. Comparing their HMS–PCI data set with interactions reported in the literature indicated that this approach was on average three times more successful at detecting known complexes than large-scale two-hybrid studies. They assert that, given the high degree of connectivity observed in their study, even partial HMS-PCI coverage of complex proteomes

Copyright © 2002 John Wiley & Sons, Ltd. 

(including that of humans) should allow comprehensive identification of cellular networks.

On March 15th, a team from Yale University reported on a **large-scale protein localisation study in *Saccharomyces cerevisiae*** (Kumar *et al.*, 2002). They epitope-tagged 60% of the yeast proteome and then used high-throughput immunolocalisation of the tagged gene products to determine the subcellular localization of 2744 proteins, 955 of which are of previously unknown function. Extrapolating these data to cover the entire yeast 'localizome' (the subcellular distribution of all 6100 yeast proteins) gave $\sim$5100 soluble proteins and $>$1000 transmembrane proteins. Their results indicate that 47% of yeast proteins are cytoplasmic, 13% are mitochondrial, 13% are exocytic (including proteins of the endoplasmic reticulum and secretory vesicles), and 27% are nuclear or nucleolar. A searchable database including 2900 fluorescent micrographs is available at: **http://ygac.med.yale.edu/**.

In the April issue of *Nature Genetics*, Klose *et al.* (2002) reported on a **genetic analysis of the mouse brain proteome**. They resolved 8767 proteins by large-gel two-dimensional electrophoresis and detected 1324 polymorphic proteins from the European collaborative interspecific backcross. Of these, 665 were mapped genetically and 466 were identified by mass spectrometry. Qualitatively polymorphic proteins, to 96%, reflected changes in conformation and/or mass. Quantitatively polymorphic proteins showed a high frequency (73%) of allele-specific transmission in codominant heterozygotes. They found that variations in protein isoforms and protein quantity often mapped to chromosomal positions other than that of the structural gene, indicating that single proteins may act as polygenic traits. They suggest that this approach may detect the types of polymorphism that are most relevant in disease-association studies.

### Transcriptomics

In the April 2nd issue of *PNAS*, a group from The Scripps Research Institute and The Genomics Institute of the Novartis Research Foundation published a large-scale study of the **human and mouse transcriptomes** (Su *et al.*, 2002). They have profiled gene expression from 91 human and mouse samples from a range of tissues, organs, and cell lines. Since the samples predominantly represent the normal physiological state, they assert that their dataset forms a preliminary, but substantial, description of the normal mammalian transcriptome. Their data provides insights into molecular and physiological gene function, mechanisms of transcriptional regulation, disease etiology, and comparative genomics. Their data is available at **http://expression.gnf.org**. The site integrates data visualization and curation of current gene annotations.

### Genomics in medicine

In the February 16th issue of *The Lancet*, Petricoin *et al.* (2002) published the results of a **trial of the use of proteomic patterns in serum for identifying ovarian cancer**. The group used SELDI-TOF mass spectroscopy to analyse low-molecular-weight serum proteins. Using spectra from a training set of 50 unaffected and 50 ovarian cancer patients, their iterative searching algorithm identified a proteomic pattern that completely discriminated the two groups. When applied to a masked set comprising 50 ovarian cancer patients and 66 women who were unaffected or had non-malignant disorders, the algorithm correctly recognised all of the cancer patients (including those at stage I) and 63 of the non-malignant cases. These results give a sensitivity of 100%, a specificity of 95% and a positive predictive value of 94%. The method requires a small sample, such as could be collected by a fingerprick, and takes around 30 minutes.

### Funding

The **National Science Foundation** has announced a research grant competition aimed solely at young investigators interested in plant genomics. A statement released on December 28th, stated that the program, called YIA-PGR, 'seeks to increase participation of young scientists in [NSF's] plant genome research, especially those at institutions that have not participated in its plant genome research program in the past' (NSF statement Dec. 28th, 2001). Academic researchers who earned their PhD on or after January 1, 1997, and hold tenure or nontenure track positions will be eligible. That includes postdoctoral fellows with an independent research program at institutions that allow them to serve as a principal investigator on a grant. Postdoctoral researchers who are about to assume a faculty position may also apply.

Copyright © 2002 John Wiley & Sons, Ltd.

On the 18th March the **European Commission** announced that they had awarded 39.4 million to three large-scale genomics projects (European Commission statement Mar. 18, 2002). The three projects are Studies of European volunteer twins to identify genes involved in common diseases, co-ordinated by Prof. Leena Peltonen (Helsinki, Finland), Understanding human disease through mouse genomics, co-ordinated by Prof. Steve Brown (Harwell, UK) and Prof. Pierre Chambon (Strasbourg, France), and Structural proteomics in Europe, co-ordinated by Prof. David Stuart (Oxford, UK) and Prof. Dino Moras (Strasbourg, France). This funding is a prelude to the 2.2 billion that has been earmarked for 'genomics research for human health' in the forthcoming Sixth Framework Programme (2002–2006).

Upstream is a compilation of brief reports on papers and press releases of interest to our readers. They represent a personal critical analysis of the original content. If you would like to recommend a paper, or newsworthy item, please contact our Managing Editor.

### References

Akerley BJ, Rubin EJ, Novick VL, *et al.* 2002. A genome-scale analysis for identification of genes required for growth or survival of *Haemophilus influenzae*. *Proc Natl Acad Sci U S A* **99**: 966–971.

Butler D. 2002. Geneticists get steamed up over public access to rice genome. *Nature* **416**: 111.

Dziejman M, Balon E, Boyd D, *et al.* 2002. Comparative genomic analysis of *Vibrio cholerae*: genes that correlate with cholera endemic and pandemic disease. *Proc Natl Acad Sci U S A* **99**: 1556–1561.

European Commission statement Mar. 18, 2002: **http://europa.eu.int/comm/research/press/2002/pr1803en.html**

Fitz-Gibbon ST, Ladner H, Kim U-J, *et al.* 2002. Genome sequence of the hyperthermophilic crenarchaeon *Pyrobaculum aerophilum*. *Proc Natl Acad Sci U S A* **99**: 984–989.

Gavin A-C, Bösche M, Krause R, *et al.* 2002. Functional organization of the yeast proteome by systematic analysis of protein complexes. *Nature* **415**: 141–147.

Goff SA, Ricke D, Lan T-H, *et al.* 2002. A draft sequence of the rice genome (*Oryza sativa* L. ssp. *japonica*). *Science* **296**: 92–100.

Ho Y, Gruhler A, Heilbut A, *et al.* 2002. Systematic identification of protein complexes in *Saccharomyces cerevisiae* by mass spectrometry. *Nature* **415**: 180–183.

International Human Genome Sequencing Consortium. 2001. Initial sequencing and analysis of the human genome. *Nature* **409**: 860–921.

Klose J, Nock C, Herrmann M, *et al.* 2002. Genetic analysis of the mouse brain proteome. *Nat Genet* **30**: 385–393.

Kumar A, Agarwal S, Heyman JA, *et al.* 2002. Subcellular localization of the yeast proteome. *Genes Dev* **16**: 707–719.

Myers EW, Sutton GG, Smith HO, Adams MD, Venter JC. 2002. On the sequencing and assembly of the human genome. *Proc Natl Acad Sci U S A* **99**: 4145–4146.

NSF statement Dec. 28th, 2001: **http://www.nsf.gov/pubs/2002/nsf02048/nsf02048.txt**

Paddison PJ, Caudy AA, Hannon GJ. 2002. Stable suppression of gene expression by RNAi in mammalian cells. *Proc Natl Acad Sci U S A* **99**: 1443–1448.

Petricoin EF, Ardekani AM, Hitt BA, *et al.* 2002. Use of proteomic patterns in serum to ifdentify ovarian cancer. *Lancet* **359**: 572–577.

PlasmoDB: **http://www.plasmodb.org/**

Salanoubat M, Genin S, Artiguenave F, *et al.* 2002. Genome sequence of the plant pathogen *Ralstonia solanacearum*. *Nature* **415**: 497–502.

Sanger *M. bovis* page: **http://www.sanger.ac.uk/Projects/M_bovis/**

Shimizu T, Ohtani K, Hirakawa H, *et al.* 2002. Complete genome sequence of *Clostridium perfringens*, an anaerobic flesh-eater. *Proc Natl Acad Sci U S A* **99**: 996–1001.

Su AI, Cooke MP, Ching KA, *et al.* 2002. Large-scale analysis of the human and mouse transcriptomes. *Proc Natl Acad Sci U S A* **99**: 4465–4470.

Venter JC, Adams MD, Myers EW, *et al.* 2001. The sequence of the human genome. *Science* **291**: 1304–1351.

Waterston RH, Lander ES, Sulston JE. 2002. On the sequencing of the human genome. *Proc Natl Acad Sci U S A* **99**: 3712–3716.

Wixon J. 2002. Featured Organism: *Schizosaccharomyces pombe*, the fission yeast. *Comp Funct Genom* **3**: 194–204.

Wixon J, Dicks J. 2002. Meeting Highlights: Plant, Animal and Microbe Genomes X. *Comp Funct Genom* **3**: 178–193.

Wood V, Gwilliam R, Rajandream MA, *et al.* 2002.The genome sequence of *Schizosaccharomyces pombe*. *Nature* **415**: 871–880.

Yu J, Hu S, Wang J, *et al.* 2001. A draft sequence of the rice (*Oryza sativa* ssp.*indica*) genome. *Chinese Science Bulletin* **46**: 1937–1942.

Yu J, Hu S, Wang J, *et al.* 2002. A draft sequence of the rice genome (*Oryza sativa* L. ssp. *indica*). *Science* **296**: 79–92.

Copyright © 2002 John Wiley & Sons, Ltd. 
